# Vicarious Dissonance: Pre-Registered Meta-Analysis

**DOI:** 10.1177/01461672241266653

**Published:** 2024-08-10

**Authors:** Sara Jaubert, Adrien Alejandro Fillon, Lionel Souchet, Fabien Girandola

**Affiliations:** 1 Aix Marseille University, Aix-en-Provence, France; 2 University of Cyprus, Nicosia, Cyprus

**Keywords:** vicarious cognitive dissonance, meta-analysis, pre-registration, attitude, hypocrisy judgments, discomfort

## Abstract

The vicarious cognitive dissonance process predicts that observing an inconsistent act by a member of the ingroup causes uncomfortable arousal in the observer, inducing a motivation to reduce this discomfort. This meta-analysis examined the effect of vicarious cognitive dissonance based on 24 studies (*N* = 16,769). Our results indicated a small effect for the vicarious cognitive dissonance (*g* = 0.41 [0.27, 0.54], *p* <.001) with important variability between the outcomes. Our moderator analysis was limited by the low number of included studies. Publication bias analyses indicate a small true effect size (e.g., 3PSM: *g* = 0.22, *p* = .042), that was inflated by small sample sizes (R-index = 14.6%). We discussed theoretical issues concerning the psychological processes underlying vicarious cognitive dissonance, and methodological questions concerning operationalization. We proposed ways of improving the design and procedure to ensure that the effects found in the literature exist and are replicable.

## Introduction

Almost 70 years ago, Festinger (1957)introduced the cognitive dissonance theory, a fundamental principle in social cognition (Gawronski & Strack, 2012). This theory focuses on situations in which individuals are faced with an inconsistency between two of their cognitions. This inconsistency causes an uncomfortable state of arousal leading the individual to engage in cognitive work oriented toward its reduction or elimination. Since then, a tremendous amount of work has been conducted to study this personal cognitive dissonance process (Fointiat et al., 2013; Priolo et al., 2019; Vaidis & Bran, 2019). Different means have been used to create situations in which an individual can experience cognitive dissonance. The three main paradigms are the free-choice paradigm (Brehm, 1956), the induced-compliance paradigm (Festinger, 1957), and the induced-hypocrisy paradigm (E. Aronson et al., 1991). The necessary conditions for the cognitive dissonance process to happen are still debated (Bran & Vaidis, 2022; Harmon-Jones & Harmon-Jones, 2019, 2022; Vaidis & Bran, 2019). While proponents of cognitive dissonance theory broadly agreed with Festinger’s (1957) theory, they differed on how to explain cognitive dynamics. Some authors considered that cognitive dissonance required triggers such as self-concept (J. Aronson et al., 1999; Leippe & Eisenstadt, 1999; Steele, 1988; Stone & Cooper, 2001), personal responsibility (Cooper, 2007; Wicklund & Brehm, 1976), social norms (Cooper & Fazio, 1984), or behavioral commitment (Beauvois & Joule, 1999). The replication crisis also affected the field of cognitive dissonance. Issues related to its nature, the necessary conditions for its occurrence, or the difficulty of its measurement were found and discussed (Bran & Vaidis, 2022; Vaidis & Bran, 2019). Several researchers in the field of cognitive dissonance theory conducted thoughtful studies aimed at best addressing these methodological limitations; one example is the pre-registered multi-laboratory replication project (Vaidis et al., 2023).

### A New Form of Dissonance

**Figure 1.**
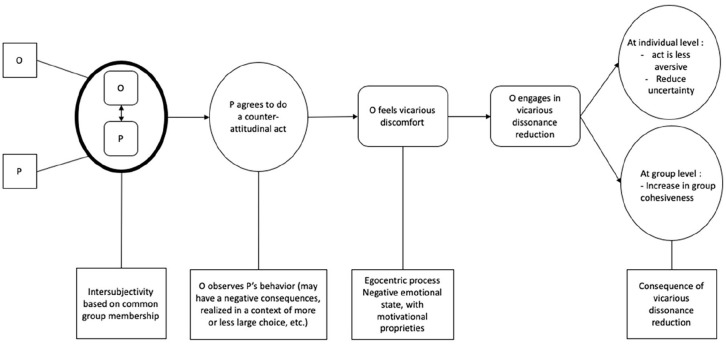
A Model of Vicarious Cognitive Dissonance (Jaubert et al., 2020). *Note*. O = observer; P = observed person.

Although most studies on cognitive dissonance have focused on personal cognitive dissonance, some have suggested that dissonance can be influenced by an individual’s membership in a particular social group (Glasford et al., 2009; Matz & Wood, 2005; McKimmie et al., 2009). More recently, a new form of dissonance has been explored, namely, vicarious cognitive dissonance, explicitly based on group membership (see Jaubert et al., 2020, for an overview, see Figure 1). Norton and colleagues (2003) investigated the vicarious experience of cognitive dissonance. In their Study 1, the authors examined the consequences of one participant observing another student agreeing with a position that the observer considered counter attitudinal. They created a fictitious cover story in which they asked Princeton University students to observe a fellow student agreeing to make a speech in favor of raising tuition. This discourse went against the students’ view. The authors measured the effects of this situation on the participants’ attitudes toward raising tuition. The participants changed their attitudes when they heard that the other student made a counter-attitudinal speech, but only if they strongly identified with their group. This study is the first publication indicating that cognitive dissonance can appear not only for a personal action but also when observing an ingroup member doing an action.

### The Theory Underlying Vicarious Cognitive Dissonance

According to social identity (Tajfel & Turner, 1979) and self-categorization theories (Turner et al., 1987), individuals represent groups in terms of prototypes defined by a set of attributes such as attitudes, intentions, beliefs, perceptions, feelings, or behaviors. These prototypes describe and prescribe the attributes of the group and allow individuals to divide their social world into distinguishable categories (Hamilton & Sherman, 1996). They can then accentuate the differences between ingroup and outgroup members (Turner et al., 1987). Categorization is related to depersonalization; it changes a subject’s social perception of self, named self-categorization, and others, named more broadly categorization. The perception of a single individual is replaced by a relevant prototype of the ingroup or outgroup, shifting the focus from differences between the self and others to a profile of prototypical similarity or interchangeability within the group. This categorization process creates intersubjectivity between ingroup members (Turner et al., 1987). When group membership is salient, and the individual is strongly identified with their group, they can share the same emotional experience (Mackie et al., 2009). In other words, if we belong to the same group, we feel the same way.

This process can lead to vicarious cognitive dissonance. If an individual feels they belong to a group with which they share emotions, they can experience a cognitive dissonance vicariously when they witness an inconsistent act committed by another member of the group (Hogg & Cooper, 2006; Monin et al., 2004; Norton et al., 2003). The negative arousal produced by this situation has been studied using self-reported discomfort measures (cognitive dissonance thermometer; Elliot & Devine, 1994) or measures designated to test the reduction of the dissonance, such as attitudinal or behavioral change (Focella et al., 2016; Norton et al., 2003).

### The Rising Interest in Vicarious Cognitive Dissonance Theory

The vicarious cognitive dissonance has important practical implications, especially for attitudes and behavior change in the areas of health (Focella et al., 2016), environment (Gaffney et al., 2012), and road safety (Barden et al., 2013). If personal cognitive dissonance manipulations seem to be effective (Priolo et al., 2019), they require effort and time to generate dissonance effects in individuals (Focella et al., 2016). Such costs might be too important for use in prevention campaigns which aim to quickly influence the attitudes or behaviors of large audiences. Vicarious cognitive dissonance has the potential to overcome these limitations by providing an effective means of influencing attitudinal and behavioral change as well as reaching a larger, geographically dispersed audience (Cooper, 2019; Focella et al., 2016).

### A Meta-Analysis on Vicarious Cognitive Dissonance

This study is a meta-analysis of vicarious cognitive dissonance. Our core questions are: How strong is the main effect of vicarious cognitive dissonance on affect, hypocrisy judgments, and attitudes? What is the estimated effect size of the vicarious cognitive dissonance? What are the important factors moderating the effect of vicarious cognitive dissonance on affect, hypocrisy judgments, and attitudes?

### Main Effect of Vicarious Cognitive Dissonance

A summary of our hypotheses can be found in Table 1. We detail them below. Definition and measures of dependent variable used in the meta-analysis can be found in Table 2.

**Table 1.** Summary of Meta-Analysis Hypothesis.

Hypotheses	Key findings/theories in the literature	Findings in the meta-analysis
*Main hypothesis*	H1_a_: The presence of vicarious cognitive dissonance^1^ will cause uncomfortable arousal in the observer, resulting in a stronger personal discomfort than the control group.	Not Supported
	H1_b_: The presence of vicarious cognitive dissonance will cause uncomfortable arousal in the observer, resulting in a stronger vicarious discomfort than the control group.	Not Supported
	H2: The presence of vicarious cognitive dissonance will cause the observer to judge the protagonist as hypocrite.	Supported
	H3_a_: The presence of vicarious induced-compliance^2^ will increase attitude or will provoke an attitude change in the direction of support for the counterattitudinal issue, contrary to the control group.	Supported for between studies but not within studies
	H3_b_: The presence of vicarious induced-hypocrisy^3^ will increase attitude or will provoke an attitude change in direction of support for the normative issue, contrary to the control group.	Supported for between studies but not within studies
	H4_a_: The presence of vicarious cognitive dissonance will provoke a change in perception of the observed person’s opinion in the direction of the inconsistent act.	Insufficient data
	H4_b_: The presence of vicarious cognitive dissonance will increase the perception of the observed person’s persuasiveness and credibility of the observer.	Insufficient data
	H5_a_: The presence of vicarious cognitive dissonance will increase similarity with the group of the observer.	Supported
	H5_b_: The presence of vicarious cognitive dissonance will increase similarity with the observed individual of the observer.	Insufficient data
Theoretical Moderator hypotheses	H6: Group membership of the observed person: the vicarious cognitive dissonance effect should be stronger when the observed person belongs to the ingroup compared to when the observed person belongs to the outgroup.	Partially supported
	H7: Choice of the observed person: the vicarious cognitive dissonance effect should be stronger when the observed person performs his act under condition of high choice compared to condition of low choice.	Not Supported
	H8: Perspective-taking priming: the vicarious cognitive dissonance effect should be stronger for an egocentric perspective compared to an “other” or an absence perspective.	Not Supported

**Table 2.** Definition and Measures of Dependent Variable in the Meta-Analysis.

Variables	Categories	Literature and hypotheses
Attitude/attitude change	Measure of attitude (Blackman et al., 2016; Focella et al., 2016; Gaffney et al., 2012; Jaubert et al., 2022a; Kennedy, 2020 2012; Norton et al., 2003 study 3; Voisin et al., 2014)	Measure of attitude, on Likert Scale, with different number of points across different studies
	Attitude change (Norton et al., 2003, study 1 and 2; Monin et al., 2004)	Subtraction of pre-experimental and post-experimental attitude, on Likert Scale
Vicarious discomfort	Level of vicarious discomfort (Blackman et al., 2016)	Measure of self-reported vicariously experienced discomfort (not reported)
	Level of vicarious discomfort (Focella et al., 2016)	Participants were next asked to respond to three statements that began, “If I were the speaker, I would have felt” . . . “uncomfortable,” “bothered,” and “uneasy,” on 9-point scale
	Level of vicarious discomfort (Norton et al., 2003, study 3; Monin et al., 2004)	Participants were asked to respond to each question (uncomfortable, uneasy, and bothered) describing how they thought they would feel in the speechwriter’s position
Personal discomfort	Level of personal discomfort (Blackman et al., 2016)	Measure of self-reported discomfort (not reported)
	Personal affect (Monin et al., 2004)	Psychological discomfort (uneasy, uncomfortable, bothered) with a personal affect perspective (. . . how you feel right now)
	Current affective state (Norton et al., 2003)	Measure of global psychological discomfort (uncomfortable, uneasy, and bothered) and self-directed negative affect (angry with myself, dissatisfied with myself, disgusted with myself, and annoyed with myself), on 7-point scale
Hypocrisy judgment	Perceived hypocrisy (Barden et al., 2005, 2013)	First three traits that came to participant’s mind regarding the observed person (coded 0 = no hypocrisy, and 1 = hypocrisy if mentioned)
	Hypocrisy judgments (Barden et al., 2005, 2013)	Measure of hypocrisy judgments, on 7-point scale
	Hypocrisy index (Barden et al., 2005, 2013)	Measure of perceived hypocrisy and hypocrisy judgments, standardized and averaged to form a hypocrisy index
Observer’s perception of observed individual’s opinion change	Assessment of observed individual’s attitudes (Barden et al. 2005, 2013; Kennedy, 2020; Monin et al., 2004)	Measure of perception of observer’s attitude, on Likert Scale, with different number of points across different studies
Observer’s perception of observed individual’s persuasiveness and credibility	Participants’ impressions of the speech (Focella et al., 2016; Norton et al., 2003)	Several items designed to assess participants’ impressions of the speech, on Likert Scale, with different number of points across different studies
Similarity with the group	Jaubert et al. (2020, 2022b)	Scale of similarity with the group of 4 items, on a 7-point scale (Stephan et al., 2011)
Similarity with the observed individual	Monin et al. (2004)	How similar they thought they were to the speaker (not reported)

Vicarious cognitive dissonance predicts that observing an ingroup member perform an inconsistent act causes an uncomfortable state of arousal in the observer who tries to reduce it (Jaubert et al., 2020; Norton et al., 2003). Some studies used the affective component of vicarious cognitive dissonance using self-reported affect scales (inspired by the cognitive dissonance thermometer; Elliot & Devine, 1994). This affective component was examined using personal discomfort, which arises from emotional contagion when witnessing the observed individual’s discomfort (Hypothesis 1_a_), and the vicarious discomfort, produced by sympathy when imagining how one would feel in the observed individual’s shoes (Hypothesis 1_b_).

Seeing a counter-attitudinal situation may lead observers to judge the observed person as a hypocrite. For example, in the induced-hypocrisy paradigm, participants are asked to establish a personal standard in front of an audience before reflecting on past private behaviors that are inconsistent with that standard and to share this inconsistency with others. This incoherence should, in theory, be negatively seen by others, leading to being judged as more hypocritical (Barden et al., 2005, 2013; Hypothesis 2).

In the vicarious induced-compliance paradigm, the participant is asked to observe an act going against their own personal standards or social norms (Norton et al., 2003). Dissonance reduction is achieved by changing their attitude toward the action performed by the observed person. The main idea of this paradigm is that people change their attitudes to accommodate the counter-attitudinal behavior of those with whom they identify. This vicarious cognitive dissonance reduction via attitude change reduces the discomfort of vicariously experienced dissonance, but it also reflects a “collective event that, we might speculate, would have a particularly powerful effect on group life [. . .] will create a general atmosphere of enhanced harmony and cohesion within the group” (Cooper & Hogg, 2007, p. 384, see Hypothesis 3_a_). In the vicarious induced-hypocrisy paradigm, members of a group support a hypocrite member of the ingroup by strengthening their beliefs in the hypocrite’s message and by taking actions that align with the hypocrite’s advice (Focella et al., 2016, see Hypothesis 3_b_).

The attitude change following negative arousal is not necessarily a way for the observer to reduce his dissonance. It can also reflect, in reference to research on social identity and conformity, a means for observers to express opinions consistent with the group norm (Abrams & Hogg, 1990; Turner & Oakes, 1989). In this case, the observer will change their attitude based on their observation because they want to conform to the attitude expressed by the observed (Hypothesis 4_a_). Due to the increase in conformity, the observer will perceive that the observed person is persuasive and credible (Hypothesis 4_b_; Focella et al., 2016; Norton et al., 2003).

Reducing dissonance could also serve the function of providing consensual validation of members’ social identity in the group and reduce feelings of self-conceptual uncertainty that might have been raised by the dissonance state (Cooper & Hogg, 2007). It might reinforce identification and solidarity within the group, which can be observed with an increase in the perception of similarity within the group (Hypothesis 5_a_; Jaubert et al., 2020, 2022b), or an increase in the observer’s perception of similarity with the observed individual (Hypotheses 5_b_; Monin et al., 2004).

### Moderators

We sought to investigate the moderators that can influence the effect of vicarious cognitive dissonance. We distinguished two types of moderators: the confirmatory moderators (see Table 3), based on hypotheses from results of previous studies, and the exploratory moderators, which emerged during the meta-analysis (see Table 4 and Table A in the Supplementary Material document).

**Table 3.** Definition and Measures of Confirmatory Moderators in the Meta-Analysis.

Variables	Categories	Literature and hypotheses
Group membership to the observed person	Categorical Variable: ingroup and outgroup	The experimenter informed that the observed person was either from their ingroup (same residential college) or outgroup (different residential college).
Observed individual’s choice	Categorical Variable: high and low	In the *high choice condition*, the experimenter left the choice to the observed person to carry out the counter-attitudinal act. In the *low choice condition*, the experimenter did not leave the choice.
Perspective-taking priming	Categorical Variable: egocentric and other	Participants are asked to respond to a scale of affect through an egocentric perspective, or other person (observed individual).

**Table 4.** Definition and Measures of Exploratory Moderators in the Meta-Analysis.

Variables	Categories	Literature and hypotheses
Type of paradigm	Categorical Variable: induced-compliance, induced-hypocrisy and free-choice	Vicarious cognitive dissonance has been studied through different paradigms (induced-compliance; induced-hypocrisy; free-choice). We will explore if there is a possibility for an effect of paradigm on dissonance.
Type of group membership	Categorical Variable: we identified 7 different group memberships	Vicarious cognitive dissonance has been studied through different group membership. We will investigate if we find stronger or weaker effect of dissonance depending on the group membership used in the scenarios.
Topics	Categorical Variable: we identified 11 different topics	Vicarious cognitive dissonance has been studied through different topics. We will investigate if we find stronger or weaker effects of dissonance depending on the topic used in the scenarios.
Type of participants	Categorical Variable: student vs. no-student	According to Henrich et al. (2010), samples of people drawn from Western, educated, industrialized, rich, and democratic (WEIRD) societies are “the least representative population one could find for generalizing about humans” (p. 61). In addition, Sears (1986) describes student participants as “a very narrow data base” (p. 527). We wanted to test the impact of type of participant on the effect of vicarious cognitive dissonance.
Type of study	Categorical Variable: online and laboratory studies	Some studies suggest that online participation is no greater a concern than those conducted in laboratory, online studies require “sensible safeguards and manipulation checks” (Casler et al., 2013, p. 2156). We wanted to test the impact of the type of study by comparing online versus laboratory studies.
Country	Categorical Variable: French, United States, and Australia	Some studies have focused on the possible cultural specificity of cognitive dissonance. About the classic distinction described in Hofstede’s (1980) work between individualistic and collectivist cultures, Miller (1984) tested the hypothesis that cognitive dissonance reduction could be a culturally determined process, and more particularly present in individualistic cultures (e.g., America, Western Europe, or Australia), rather than a universal process. Thus, we wanted to test the impact of a country on the effect of vicarious cognitive dissonance.

The process by which group members experience the same emotions as other members might be facilitated when individuals share a feeling of belonging to the same group (Mackie et al., 2009). Studies on vicarious cognitive dissonance suggest that group membership can increase the effect of vicarious cognitive dissonance (see confirmatory Hypothesis 6; Hogg & Cooper, 2006; Monin et al., 2004; Norton et al., 2003).

Dissonance is more important when the individual is responsible—when they perform an act of free choice (Cooper, 1971; Wicklund & Brehm, 1976). In the absence of personal responsibility, inconsistent situations are psychologically irrelevant to the individual, and therefore, would not generate dissonance. We hypothesize that the effect of vicarious cognitive dissonance is accentuated when the observed person performs the act with more freedom compared to having less possibilities to choose (see confirmatory Hypothesis 7).

Dissonance theorists have considered that the process leading to an attitude change in a group situation is fundamentally egocentric (Blackman et al., 2016; Keller, 2015). Thus, the vicarious cognitive dissonance effect should be accentuated in the egocentric perspective, by imagining what would happen if the person wrote the essay or did the speech compared to other perspectives, only watching the other write an essay or make a speech (see confirmatory Hypothesis 8; Blackman et al., 2016).

During our literature review, we included six exploratory moderators. We tested the moderating effect of the paradigm used (induced-compliance; induced-hypocrisy; free-choice), the topics of the scenario used, the type of the group membership used in the scenario, as well as the type of participants, type of study, and country.

## Methods

We pre-registered the data set and analysis script, shared the final data set and script with the output on OSF: https://osf.io/t5vs7/?view_only=e9dc20e90a584afbb2456aecd8809c9b.

### Literature Search

**Figure 2.**
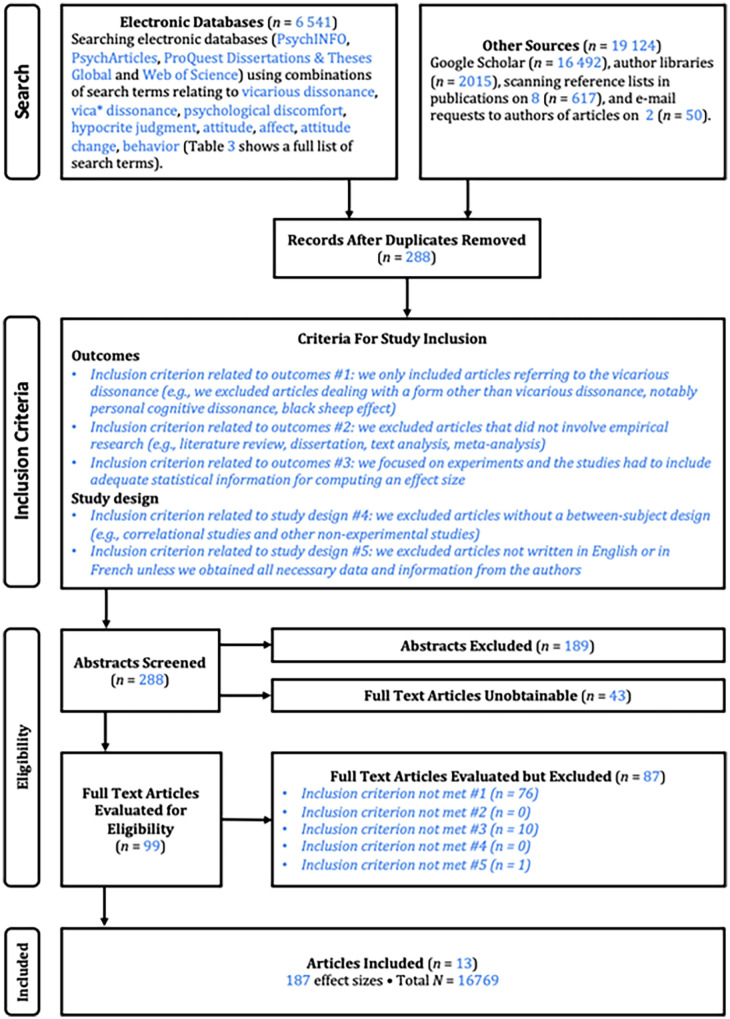
Meta-Analysis Flow Diagram Adapted Based on Moreau and Gamble (2020) and Yeung et al. (2020).

Several databases (i.e., Google Scholar, ProQuest, Web of Science, PsycINFO, and PsycArticles) were used to find articles relevant to our topic (see Table 5; Gehanno et al., 2013; Martín-Martín et al., 2018; Walters, 2007). We identified a sample of studies based on various steps illustrated in Figure 2. We then conducted a Boolean search with keywords, such as “vicarious dissonance,” “cognitive dissonance,” and “social identity” (see Table 5), which allowed us to identify relevant literature, related topics, and works in this field. We decided to not restrict the searching terms to vicarious dissonance, as we found that some studies on vicarious dissonance were conducted without using the term, such as the one from Barden and colleagues (2005). The full Boolean search can be found in the supplement and the Excel file in the “Search terms” tab (https://osf.io/t5vs7/?view_only=e9dc20e90a584afbb2456aecd8809c9b).

**Table 5.** Search Syntax, Date of Searches, and Number of Results.

Database	Date of search	Search syntax	Results
Google Scholar	11 May 2021	1	322
	19 May 2021	2	16 100
	20 May 2021	3	26
	21 May 2021	4	44
ProQuest Disserta	19 May 2021	1	51
	26 May 2021	2	6 122
	26 May 2021	3	2
	26 May 2021	4	5
Web of Science	19 May 2021	1	12
	25 May 2021	2	20
	25 May 2021	3	1
	25 May 2021	4	0
PsycINFO and PsycArticles	11 May 2021	1	29
	25 May 2021	2	292
	11 May 2021	3	7
	26 May 2021	4	0

*Note.* Four search terms were used: (a) “vicarious dissonance” OR “vica* dissonance” AND “psychological discomfort” OR “hypocrite judgment” OR “attitude” OR “affect” OR “attitude change” OR “behavior.” (b) “cognitive dissonance” AND “social identity” AND “psychological discomfort” OR “hypocrite judgment” OR “attitude” OR “affect” OR “attitude change” OR “behavior.” (c) “dissonance vicariante” AND “inconfort psychologique” OR “jugement hypocrite” OR “attitude” OR “affect” OR “changement d’attitude” OR “comportement” and (d) “dissonance cognitive” AND “identité sociale” AND “inconfort psychologique” OR “jugement hypocrite” AND “attitude” OR “affect” OR “changement d’attitude” OR “comportement.”

This process was followed by a review of the bibliographic references of the articles found, letting us identify additional articles not detected by the Boolean search procedure. We also searched for “related articles” and “cited by” Google Scholar options based on the articles found. All database searches achieved 25 665 hits (*n* = 6 541 for electronic databases; *n* = 19 124 for other sources, including Google Scholar). We decided to stop the search process after 30 consecutive not includable results based on the title, leading to 368 studies. After deleting duplicates, 288 articles remained to be assessed based on our eligibility criteria. The date of the last search was May 26, 2021.

We contacted authors who published on the topic and used ResearchGate, Twitter, and mailing lists to ask for published and unpublished data (see the mailing template in the Supplementary Material document). We received one article not previously found in our search. Based on our inclusion criteria after reading the abstract, a total of 99 published articles, unpublished articles, and data sets were initially identified and downloaded from the primary database search.

### Inclusion and Exclusion Criteria

We only included articles referring to vicarious cognitive dissonance. We excluded articles manipulating another form of dissonance such as personal cognitive dissonance, the black sheep effect, and articles that did not involve empirical research such as literature reviews or dissertations, or without a between-subjects design. Among the 99 full text articles evaluated for eligibility, 76 articles were excluded on this first criterion of exclusion.

Second, we focused on experiments, and the studies had to include adequate statistical information for computing an effect size. In cases of missing statistical data, we first attempted to contact the authors. If we were not able to obtain the required statistics, we either imputed data or excluded the articles, with the decision made available in our datasheet (see “Article list+Inc-Exc Criterion,” https://osf.io/t5vs7/?view_only=e9dc20e90a584afbb2456aecd8809c9b). Among the 99 full-text articles evaluated for eligibility, 10 articles were excluded because they did not include adequate statistical information for computing an effect size.

Finally, we excluded articles not written in English or French, unless we had obtained all necessary data and information from the authors. One article was excluded because it was not written in English or French. The reasons for exclusion are detailed in Table B in the Supplementary Material document. Finally, 13 articles including 24 unique experiments were integrated in our data set (see Table 6 for a list of included articles).

**Table 6.** Studies Included in the Meta-Analysis.

Study number	Study	*k*	Country	Sample population	Publication status	Vicarious dissonance type	DV type
1.	Barden et al. (2005)	2	United States	Students	Yes	IH	Hypocrisy judgment
2.	Barden et al. (2013)	2	United States	Students/General population	Yes	IH	Hypocrisy judgment
3.	Blackman et al. (2016)	3	United States	Students/General population	Yes	IC—FC	Attitude
4.	Focella et al. (2016)	3	Australia	Students	Yes	IH	Attitude
5.	Gaffney et al. (2012)	1	United States	Students	Yes	IH	Attitude
6.	Herak et al. (2018)	1	French	Students	Yes	Unclear	Warm/competence
7.	Jaubert et al. (2022a)	1	French	Students	No	IC	Attitude change
8.	Jaubert et al. (2022b)	1	French	Students	No	IH	Behavioral intention change
6.	Kennedy (2020)	1	United States	General population	Yes	IC	Attitude
10.	Monin et al. (2004)	2	United States	Students	Yes	IC	Attitude change
11.	Norton et al. (2003)	3	United States	Students	Yes	IC	Attitude change
12.	Voisin et al. (2014)	3	United States	Students	No	IH	Vicarious discomfort
13.	Voisin et al. (2016)	1	French	General population	No	IH	Attitude

*Note*. IH = Induced-Hypocrisy; IC = Induced-Compliance; FC = Free-Choice.

### Analyses

We ran our analysis in R Core Team (2018). Given the multiple possibilities of investigating vicarious cognitive dissonance across the studies, we expected heterogeneity in the sample to be relatively high. Therefore, a random-effects model was used. We converted all effects into a standardized effect size: Hedge’s *g*. Statistical heterogeneity was determined using the Tau-squared test and quantified using *I*^2^, which represents the percentage of the total variation in a set of studies that is actually due to between-study heterogeneity (C. A. Higgins et al., 2003). If there was indeed substantial heterogeneity, we investigated and explored potential moderators.

To analyze the moderators, we ran a meta-analysis with the moderator as predictor. Then, we conducted meta-analyses for each subgroup of the moderator. We reported the effect sizes for each subgroup, and the meta-analytical result of the difference between subgroups of the moderator.

We conducted publication bias analyses, including funnel plots and statistical tests for publication bias (publication status as a moderator, comparing effects for published findings only) and asymmetry (trim and fill, rank test, Egger’s unweighted regression symmetry test). We also conducted p-uniform, p-curve, PET, and PEESE analyses.

## Results

### Main Vicarious Cognitive Dissonance Effect

**Figure 3.**
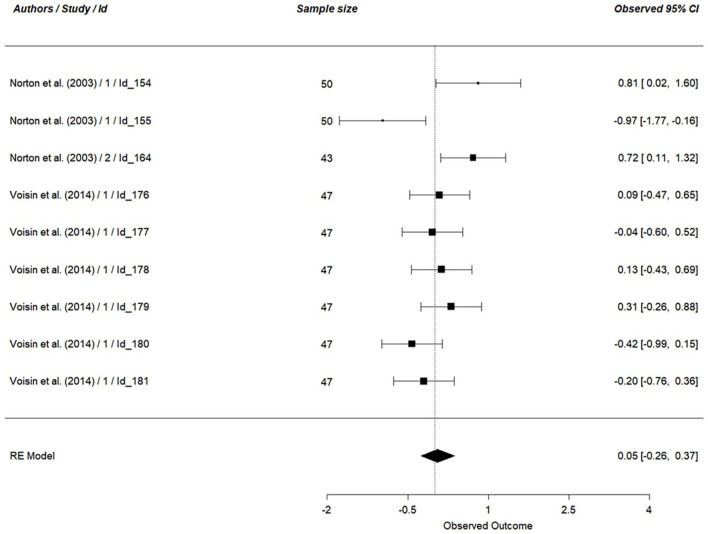
Effect of Vicarious Cognitive Dissonance on Personal Discomfort. *Note.* RE model is the averaged meta-analytical effect size Hedges’ g. The effect size of each study is indicated on the right side of the figure, along with the 95% confidence interval.

**Figure 4.**
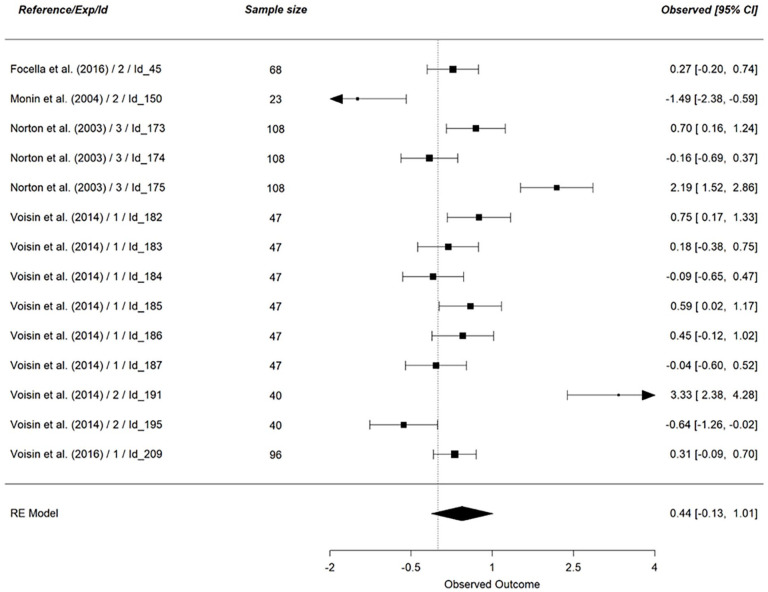
Effect of Vicarious Cognitive Dissonance on Vicarious Discomfort. *Note.* RE model is the averaged meta-analytical effect size Hedges’ g. The effect size of each study is indicated on the right side of the figure, along with the 95% confidence interval.

We first examined the main effect of vicarious cognitive dissonance (*k* = 102), on all dependent variables (see Table 7). Concerning personal discomfort, our analysis was based on 9 effect sizes (*N* = 425, see Figure 3). The results indicated no support for an effect of vicarious cognitive dissonance on personal discomfort (*g* = 0.05, 95% CI [−0.26, 0.37], *p* = .74). Looking at a scene eliciting cognitive dissonance did not lead to a stronger experience of personal discomfort. This result did not support hypothesis 1_a_. The same results hold for vicarious discomfort (*k* = 14, *N* = 873, *g* = 0.44, 95% CI [−0.13, 1.01], *p* = .13, see Figure 4), indicating that hypothesis 1_b_ was not supported by the results.

**Table 7.** Meta-Analysis Results for the Effect of Vicarious Cognitive Dissonance on the Dependent Variables.

Measure	*k*	*N*	*g*	95% CI	*Q*	*df*	*p*	*I²*
Attitude (between-subject)	23	2234	0.54	[0.30, 0.78] ***	113.66	22	< .001	86.06%
Attitude (within-subject)	35	2333	0.13	[−0.05, 0.32]	168.31	34	< .001	81.02%
Vicarious discomfort	14	873	0.44	[−0.13, 1.01]	105.261	13	< .001	92.65%
Personal discomfort	9	425	0.05	[−0.26, 0.37]	18.79	8	.02	58.14%
Hypocrisy judgments	16	1646	0.70	[0.46, 0.95] ***	75.968	15	< .001	82.61%
Similarity with the group	14	1400	0.47	[0.04, 0.91] *	131.204	13	< .001	91.87%
Combined	102	8486	0.41	[0.27, 0.54]	672.49	101	<.001	88.53%

*Note. k* = number of samples; *N* = total number of individuals in *k; g* = Hedge’s g effect size, CI = lower and upper limits of 95% confidence interval (all two-tailed).

* *p* < .05, ** *p* < .01, *** *p* <.001.

**Figure 5.**
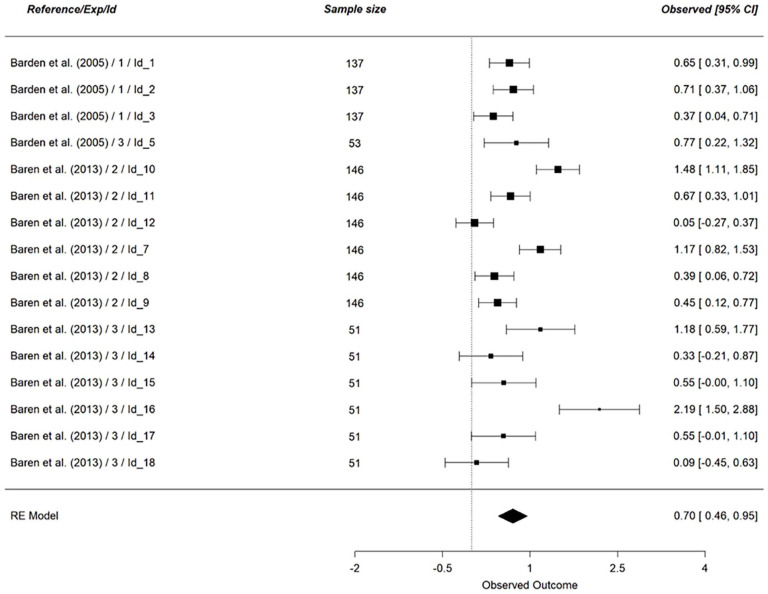
Effect of Vicarious Cognitive Dissonance on Hypocrisy Judgments. *Note.* RE model is the averaged meta-analytical effect size Hedges’ g. The effect size of each study is indicated on the right side of the figure, along with the 95% confidence interval.

On the contrary, we found a positive effect of vicarious cognitive dissonance on hypocrisy judgments (*k* = 16, *N* = 1646, *g* = 0.70, 95% CI [0.46, 0.95], *p* < .001, see Figure 5). Seeing that a cognitive dissonance scene led to judging the observed individual as more hypocritical, it is evident that this result supports hypothesis 2.

When coding the effect sizes regarding attitudes, we found that many studies measured attitudes between an experimental group and a control group, reporting the attitude after the independent variable manipulation (between-subjects); while on the other hand, others measured a change in attitude when comparing the attitude of same participants before and after the independent variable manipulation (within-subjects). Based on the gold standard methods (Carpenter, 2020), we decided to conduct a meta-analysis on each design to ensure a correct aggregation of studies.

**Figure 6.**
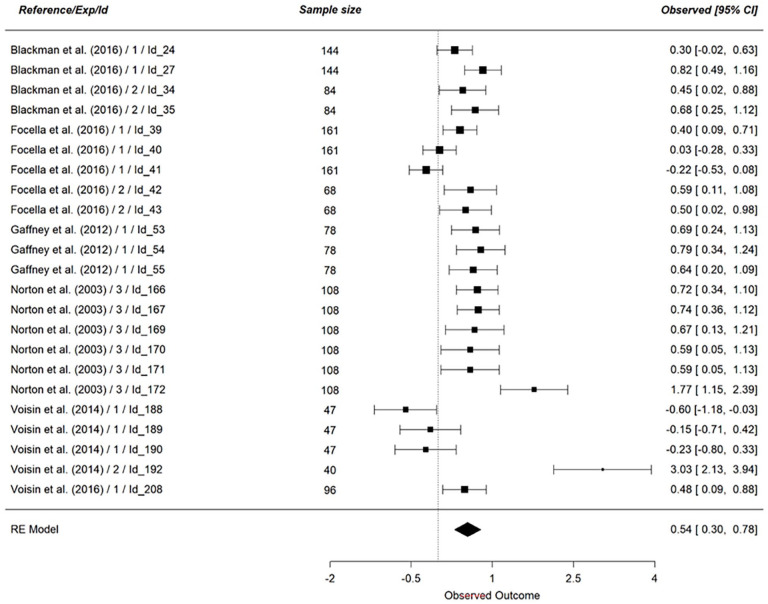
Effect of Vicarious Cognitive Dissonance on Attitude (Between-Subject). *Note.* This figure shows the aggregated estimates, but the actual analysis was conducted based on the *k*= 23 individual estimates using a multilevel model. RE model is the averaged meta-analytical effect size Hedges’ g. The effect size of each study is indicated on the right side of the figure, along with the 95% confidence interval.

**Figure 7.**
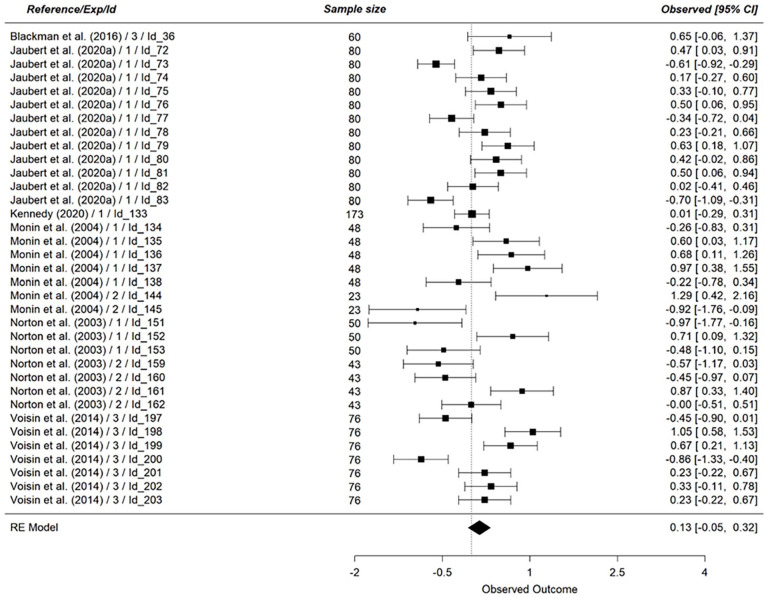
Effect of Vicarious Cognitive Dissonance on Attitude (Within-Subject). *Note.* RE model is the averaged meta-analytical effect size Hedges’ g. The effect size of each study is indicated on the right side of the figure, along with the 95% confidence interval.

Regarding the between-subject set of studies on attitudes, we found support for a moderate effect of vicarious cognitive dissonance (*k* = 23, *N* = 2234, *g* = 0.54, 95% CI [0.30, 0.78], *p* < .001, see Figure 6). For the within-subject set of studies on attitudes, we found no support for an effect of vicarious cognitive dissonance (*k* = 35, *N* = 2333, *g* = 0.13, 95% CI [−0.05, 0.32], *p* = .16, see Figure 7). Combining both results, we found mixed support regarding hypotheses 3_a_ and 3_b_.

**Figure 8.**
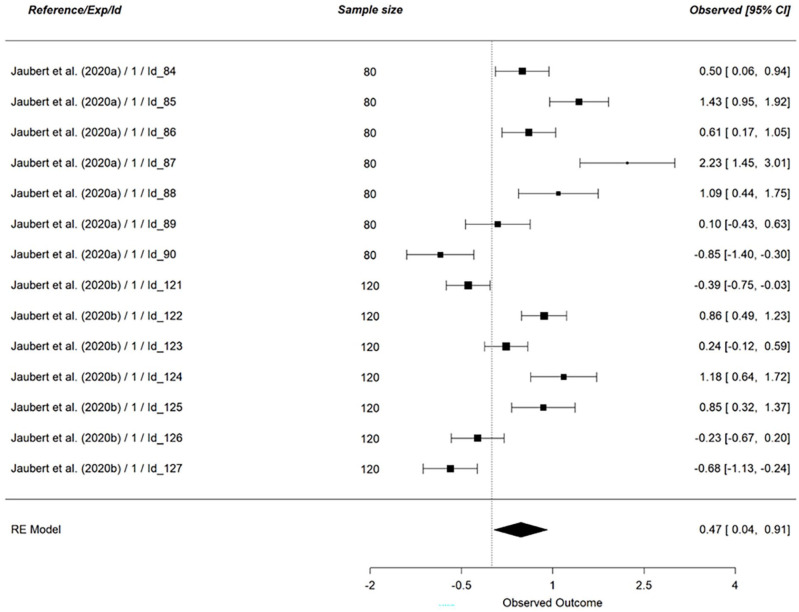
Effect of Vicarious Cognitive Dissonance on Similarity With the Group. *Note.* RE model is the averaged meta-analytical effect size Hedges’ g. The effect size of each study is indicated on the right side of the figure, along with the 95% confidence interval.

Finally, we tested if vicarious cognitive dissonance could increase the similarity with the group. Our analysis was based on 14 effect sizes (*N* = 1400; see Figure 8). Results provided support for a weak to strong effect (*g* = 0.47, 95% CI [0.04, 0.91], *p* = .03). However, we point out that the confidence interval is large, consistent with the results of heterogeneity, *Q* (13) = 131.20, *p* < .001, *I*^2^ = 91.87%. This result supports hypothesis 5_a_.

Due to a lack of sufficient data, analyses on dependent variables we collected were not conducted (i.e., similarity with the observed individual, observer’s perception of observed individual’s persuasiveness and credibility, and opinion change, see Table C in Supplementary Material document). We reported and summarized all hypotheses in Table 1 and the effect sizes in Table 7.

### Moderator Analyses

Cognitive dissonance theory suggests that cognitive dissonance would be accentuated when the observed person belongs to the ingroup rather than to the outgroup (Norton et al., 2003). We tested the importance of how belonging to the same group can increase dissonance. Results indicated that belonging to the same group (*k* = 84, *N* = 6684, *g* = 0.42, 95% CI [0.26, 0.58], *p* < .001) led to the same moderate effect of vicarious cognitive dissonance as not belonging to the observer’s group (*k* = 4, *N* = 325, *g* = 0.42, 95% CI [−0.11, 0.94], *p* = .119), since the difference between the effect sizes was not significant (*p* = 1.00).

Vicarious cognitive dissonance should be more important when the observed person acts freely, compared to a low choice condition (Norton et al., 2003). Results indicated that being freer to act (*k* = 75, *N* = 6075, *g* = 0.37, 95% CI [0.23, 0.52], *p* < .001) has the same moderate effect of vicarious cognitive dissonance as being less free (*k* = 27, *N* = 2411, *g* = 0.51, 95% CI [0.17, 0.85], *p* < .01), since the difference between the effect sizes was not significant (*p* = .46).

Finally, we tested whether the perspective taken by the observer to describe their discomfort moderated the effect of vicarious cognitive dissonance. Results indicated that an egocentric perspective (*k* = 28, *N* = 2098, *g* = 0.35, 95% CI [0.06, 0.65], *p* < .02) had the same moderate effect of vicarious cognitive dissonance (*k* = 2, *N* = 168, *g* = 0.56, 95% CI [0.26, 0.87], *p* < .001), since the difference between the effect sizes was not significant (*p* = .33).

In summary, we did not find any support for the moderators of vicarious cognitive dissonance (hypotheses 6−8 were not supported). The results are summarized in Table 8. We conducted several exploratory and inconclusive moderator analyses (i.e., effect of type of paradigm, type of participants and studies, and country) summarized in Table 9. We note that, except for the choice moderator, all include at least one condition with less than 10 estimates, with a high heterogeneity, indicating uncertainty in the findings.

**Table 8.** Summarized Results of Confirmatory Moderator Analysis.

Moderator	*k*	*N*	*g*	95% CI	*Difference*	*p*
GROUP MEMBERSHIP						
Ingroup	84	6684	0.42	[0.26, .58] ***		
Outgroup	4	325	0.42	[−0.11, 0.94]	0.001 [−0.55, 0.55]	1.00
OBSERVED’S CHOICE						
High	75	6075	0.37	[0.23, 0.52] ***		
Low	27	2411	0.51	[0.17, 0.85] **	0.14 [−0.23, 0.51]	.46
PERSPECTIVE TAKING						
Egocentric	28	2098	0.35	[0.06, 0.65] *		
Other	2	168	0.56	[0.26, 0.87] ***	0.21 [−0.22, 0.63]	.33

*Note. k* = number of samples; *N* = total number of individuals in *k; g* = Hedge’s g effect size, CI = lower and upper limits of 95% confidence interval. DVs included = attitude and attitude change, hypocrisy judgments, vicarious discomfort, and similarity to group and observed. *Difference* is the Hedges’ *g* difference between the conditions. *P*-values comes from a moderator analysis based on a Wald test comparing the effect sizes between conditions: * *p* < .05, ** *p* < .01, *** *p* <.001 (all two-tailed).

**Table 9.** Summarized Results of Exploratory Moderator Analysis.

Moderator	*k*	*N*	*g*	95% CI	*Difference*	*p*
TYPE OF PARADIGM						
Induced-Hypocrisy	53	4674	0.46	[0.27, 0.64] ***		
Induced-Compliance	48	3752	0.35	[0.14, 0.55] ***	−0.11 [−0.38, 0.17]	.43
Free-choice	1	60	0.65	[−0.06, 1.37]	−0.20 [−0.94, 0.54] −0.31 [−1.05, 0.44]^a^	.60 .42
PARTICIPANTS/STUDIES						
Students/Laboratory	85	7013	0.39	[0.25, 0.52] ***		
No students/Online	17	1473	0.54	[0.07, 1.02] *	0.16 [−0.34, 0.65]	.53
COUNTRY						
France	28	2552	0.31	[0.07, 0.55] *		
United States	68	5247	0.46	[0.28, 0.64] ***	0.15 [−0.15, 0.45]	.33
Australia	6	687	0.23	[−0.03, 0.49]	0.08 [−0.27, 0.44] 0.23 [−0.08, 0.55]^b^	.65 .15

a Free-Choice vs. Induced-Compliance. ^b^ Australia vs. United States.

### Bias

#### Publication Bias

**Figure 9.**
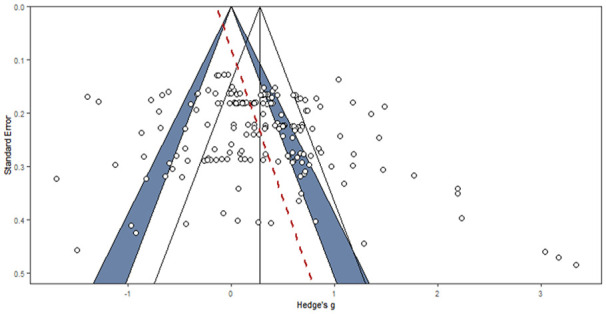
Funnel Plot. *Note.* Funnel plot displays the effect size against standard errors. If a true effect is present without publication bias, the effect size should be affected only by noise. It implies that all effect sizes should be below the pyramid, as the more error the effect size is associated with, the wider the dispersion should be around the averaged effect size. The blue pyramid corresponds to the 95% confidence interval around the meta-analytical result. The red line is the Egger test of asymmetry and indicates symmetry only if vertical. An asymmetry, as displayed in our analysis, is an indicator of publication bias.

A summary of publication bias analyses is provided in Table 10. The different tests do not indicate evidence of clear asymmetry, while the distribution of studies on the Funnel plot (see Figure 9) and Egger’s regression test indicate asymmetry (*z* = 2.34; *p* = .02; *b* = −0.19, 95% CI [−0.70, 0.33]; Harrer et al., 2021).

**Table 10.** Publication Bias Analyses Results.

Publication bias analysis method	Results and adjusted models
Egger’s regression test	*z* = 2.34, *p* = .02
Rank correlation test	*Kendall’s tau* = 0.09, *p* = .16
Trim and fill funnel plot asymmetry	0 studies missing on the left side. Adjusted Model: *g* = 0.41 [0.27, 0.54] ***
P-Curve	Evidential value: yes, P-Curve Adjusted Cohen’s *d* = 0.57
Puniform	Adjusted Model: *g* = 0.64 [0.52, 0.75] ***, 53 significant studies
Three-parameter selection model	Likelihood Ratio Test: 5.73, *p* = .02 Adjusted Model: *g* = 0.20 [−0.02, 0.41]
Henmi and Copas (2010)	Adjusted Model: *g* = 0.36 [0.23, 0.49] ***
PET	*b* = −0.02 [−0.48, 0.44], *p* = .92
PEESE	*b* = 0.173 [−0.06, 0.41], *p* = .15

*Note*. Values in brackets indicate 95% confidence intervals.

The p-curve analysis suggests that the p-value distribution curve is significantly skewed to the right, and that our data most likely contain a true underlying effect. However, given the high intra-cluster heterogeneity estimate of our effect sizes (*I*^2^ = 88.53%), the corrections to the effect size estimates proposed by the p-curve, p-uniform, PET-PEESE, and Trim and Fill methods are not the most appropriate ones, as these lose reliability as within-group heterogeneity increases (Carter et al., 2019).

The 3PSM is one of the most robust estimators of publication bias (Carter et al., 2019). It indicates a null effect of vicarious cognitive dissonance (*3PSM: g* = 0. 20, 95% CI [−0.02, 0.41], *p* = .07; *T*^2^ = 0.39). All other models indicate a non-null effect size, but lower than the ones reported in the main analysis. Altogether, these estimators lead to the conclusion that the true effect size of the vicarious cognitive dissonance might be overestimated in the literature.

**Figure 10.**
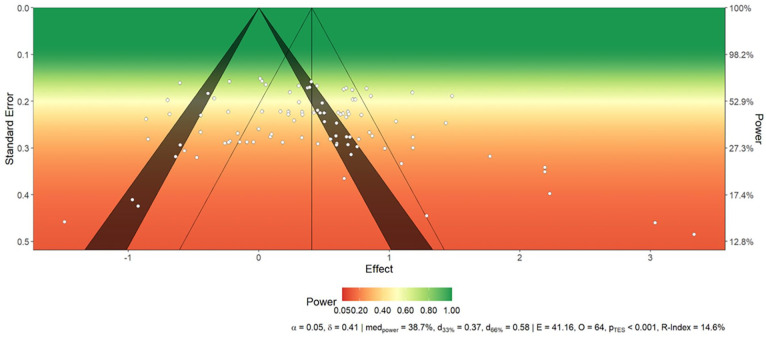
Power Analysis. *Note.* We set an alpha to 0.05. The redder the area, the less power, the greener, the more. The median power is 38.7% and replicability index is 14.6%, which means that we have less than 39 % of chance to reject H0 when there is a true effect, and 15% chance to replicate one study (see Motyl et al., 2017 for R-index).

Finally, most of the studies included in our meta-analysis have very low power relative to the effect size found in our results. With an estimated effect size of *d* = 0.41, a power of 95%, an alpha of 5%, an equal number of participants in each group, and a one-tailed test, we would need 156 participants per group to have a chance of detecting the efficacy of this effect (see Figure 10). However, none of the studies included in our meta-analysis has the necessary power to detect an effect of vicarious cognitive dissonance. More importantly, the average power in the included studies is 38.7% and the replicability index is 14.6%. This means that we have a less than 39% chance of rejecting H0 if there is a true effect, and a15% chance of replicating a study (see Motyl et al., 2017 for the replicability index).

#### Qualitative Analysis of Risk of Bias

We used the Risk of Bias 2 (ROB2, J. P. T. Higgins et al., 2019) analysis, with an estimate of the per-protocol effect, or the effect of adherence to interventions as specified in the trial protocol (Hernán & Robins, 2017), covering 5 different types of biases (i.e., bias arising from the randomization process, bias due to deviations from intended interventions, bias due to missing outcome data, bias in the measurement of the outcome, bias in the selection of the reported result).

**Figure 11.**
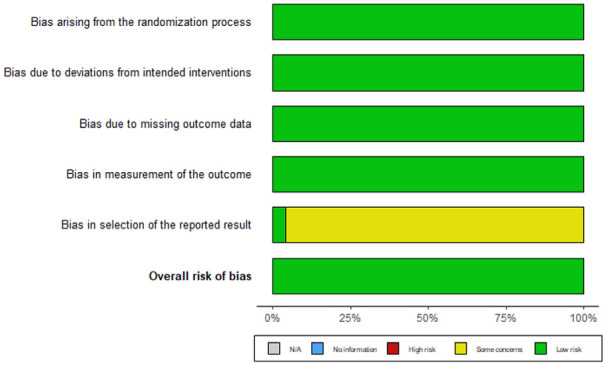
Risk of Bias Domains. *Note.* We set an alpha to 0.05. The redder the area, the less power, the greener, the more. The median power is 38.7% and replicability index is 14.6%, which means that we have less than 39 % of chance to reject H0 when there is a true effect, and 15% chance to replicate one study (see Motyl et al., 2017 for R-index).

For all studies included in our meta-analysis, our RoB2 analysis appears to show a low risk of bias for 4 of the 5 outcome domains (see Figure 11). Regarding the bias in the selection of the reported outcome, most studies are noted as concerning, but not at high risk of bias. This comes from the observation that most studies were not pre-registered.

Using only the lower risk of bias studies (*k* = 24), we found a positive effect, *g* = 0.43, 95% CI [0.27, 0.59], *p* < .001, and considerable heterogeneity, *Q* (21) = 72.37; *p* < .001; *I*^2^ = 73.99%. This qualitative assessment indicates than only a small fraction of the included effects has a low risk of bias (24/102). Increasing the quality of studies included does not seem to improve between study variation not due to sampling error.

## Discussion

We conducted a meta-analysis to examine when vicarious cognitive dissonance leads to a change in the observer, such as feeling uncomfortable, changing their attitude toward the observed person, judging them as more hypocritical, or feeling more similar to other ingroup members. Results indicated that vicarious cognitive dissonance leads to an increase in judgment of hypocrisy and a higher level of similarity with the group. Results were mixed regarding attitudes, as we found a difference in attitude in studies using a between-subject design, but not using a within-subject design. Finally, results do not indicate a relationship between vicarious cognitive dissonance and an increase in discomfort, either for personal or vicarious discomfort.

### Vicarious Cognitive Dissonance Does Not Elicit Discomfort

One of the most surprising results of our analysis is that vicarious cognitive dissonance does not elicit discomfort, either vicarious or personal, contrary to the predicted theory (Cooper & Hogg, 2007; Jaubert et al., 2020). Regarding vicarious discomfort, one simple explanation might be that in some studies (Blackman et al., 2016; Monin et al., 2004), vicarious discomfort was measured too late, after the participant successfully reduced the discomfort to return to a neutral state. We make this inference based on a set of studies on attitude change, showing that personal discomfort was reduced when measured after an attitude change, presumably because attitude change is an effective way to reduce psychological discomfort (Elliot & Devine, 1994). The same pattern could be found for vicarious discomfort (Norton et al., 2003). However, Focella et al. (2016) measured vicarious discomfort before attitudes but did not find support for an effect on vicarious discomfort. The authors indicated that it was necessary to distinguish between two situations eliciting dissonance.

In a situation of induced-compliance, the participant observes the performance of an act going against their own or their group’s values. In this situation, the counter-attitudinal act may be the source of vicarious discomfort because it is perceived by group members as a transgression of group norms. In a situation of hypocrisy, the participant observes a situation in which the observer advocates a pro-social course of action and is then asked to recall past behaviors that are contrary to that course of action. In this case, the transgression may be perceived as less obvious, because it includes a declaration consistent with the group norms. In this situation, observers are then less likely to experience vicarious discomfort.

One way of answering this debate would be to use novel designs. Since it is difficult to measure implicit psychological processes such as personal cognitive dissonance, it is possible to build designs that can manipulate this process or, as Spencer and colleagues (2005) posit, use moderation-of-process designs. Such models have already been implemented in studies on personal cognitive dissonance (Zanna & Cooper, 1974).

In addition, we note that vicarious discomfort was mostly measured with items from the Dissonance Thermometer (Elliot & Devine, 1994), initially used to measure personal discomfort, and modified as “If I were the speaker, I would have felt. . . .” However, the validity of this scale has been questioned (Lozza & Benoît, 2002). One study found dramatically low reliability of the discomfort items (Harmon-Jones, 2000; α = .48). This argument can also apply to our null finding regarding personal discomfort.

### A Complex Relationship Between Dissonance and Attitude

We found that vicarious cognitive dissonance influences attitude when comparing attitudes after the independent variable manipulation, but not when comparing attitude changes before and after the independent variable manipulation. While researchers expect participants’ attitudes to be consistent over time, several studies on dissonance seem to show a difference in attitude between pre- and post-experimental conditions, even in a control group free of dissonance (Jaubert et al., 2020; Vaidis et al., 2023). In addition, in most included studies, participants’ attitudes were measured using single-item scales (Focella et al., 2016; Monin et al., 2004; Norton et al., 2003). Attitudes are multidimensional, comprising affective, cognitive, and behavioral dimensions (Eagly & Chaiken, 2007). Thus, a single-item attitude measure assesses specific aspect of an attitude, and not necessarily all dimensions of the attitude (Martinie et al., 2013). Second, the context in which attitudes were measured, such as the time between the pre- and post-experiment measurement, the location where the questionnaire was completed, or if the research was conducted online or in a laboratory, may influence participants’ responses (Kintz et al., 1965; Marczyk et al., 2010).

We advocate for the development of a measurement of attitude change (i.e., pre- and post-experimental measures) to allow assessment of actual change in participants’ attitudes, using multidimensional scales to gain precision on the vicarious cognitive dissonance effect on attitude change.

### Uncovering the Underlying Process of Vicarious Cognitive Dissonance

This meta-analysis highlights a thinking error that should be avoided: the presence of a dissonance reduction mode (e.g., attitude change or behavior change) proves the existence of a dissonance state. Many dissonance regulation strategies exist (Voisin et al., 2013), but the predictions that can be made about the use of regulation strategies remain fairly limited (Bran & Vaidis, 2022). The presence of a dissonance regulation mode cannot guarantee the presence of a cognitive dissonance state. As the relationship is not evident anymore, it is essential to rethink the study of dissonance by focusing our efforts on developing a reliable instrument measuring the nature of the cognitive dissonance state as well as its magnitude. Physiological measures (brain activity measures, Harmon-Jones, 2003, 2004; electrodermal measures, Elkin & Leippe, 1986; Harmon-Jones et al., 1996; heart rate, Mann et al., 1969), enriched with measures such as the think-aloud method (Ericsson & Simon, 1980), could ensure a presence of a cognitive dissonance state, increasing reliability of the manipulation. Complementary methods, such as conducting debriefings or interviews at the end of the experiments, are promising alternatives to studying these processes, allowing a free expression of feelings in dissonance situations.

### The Factors Influencing Vicarious Cognitive Dissonance

We expected the effect of vicarious cognitive dissonance to be stronger when the observed individual belonged to the same group as the observer, compared to when they belonged to a different group (i.e., outgroup member condition). However, our results indicated that belonging to the same group is not necessary for the dissonance, as they lead to the same effect as with the outgroup. We note that the groups used in vicarious cognitive dissonance studies can be seen as wide (political identity, age category, university affiliation). It is possible that the “outgroup” members were perceived by the individuals as ingroup members, referring to other equally inclusive affiliations (gender, human beings) because of the vagueness of the group definition. If individuals can feel vicarious cognitive dissonance with any individual because they always share a common belonging to a group such as being human, then the latter does not seem to be a relevant moderating variable of the vicarious cognitive dissonance effect. The observer’s identification with the observed person’s group, or his or her perceived proximity to the observed person seems to be more important to explain the vicarious cognitive dissonance. Nevertheless, we are limited in our interpretation by the small number of studies in the “outgroup” condition (*k* = 4).

In their third experiment, Focella et al. (2016) observed that only participants who strongly identified with the ingroup reported a lower perception of the observed individual’s choice under the “high choice” conditions. It is as if strongly identified participants, who want the hypocrite to be honest and truthful, when observing a transgression by an ingroup member, claim that they had no choice but to transgress the norm. Norton et al. (2003) explain this lack of moderation of choice by the fundamental attribution error that, at least in Western cultures, results in an underestimation of situational constraints in attributing the behavior of others (Jones & Harris, 1967). Although choice is often considered a key variable in the induction of personal cognitive dissonance (Cooper & Fazio, 1984), participants in a vicarious cognitive dissonance situation may overestimate the observed individual’s choice of positions relative to their own. Following Norton and colleagues’ (2003) point of view, our results indicated that experimental procedures are not precise regarding the kind of choice and degree of freeness involved. In most cases, the “lower level” and “higher level” of choices are methodologically similar to each other. Indeed, the difference between the high and low choice conditions consist in a change of one sentence (addition, in the “high choice” condition of a sentence such as “your participation is completely voluntary [. . .] We would appreciate your help, but we do want to let you know that it’s completely up to you” [Vaidis et al., 2023]).

Finally, when testing the perspective taken by the participants, we found that nearly all of them approach an egocentric perspective (*k* = 2 others). This finding is not surprising as researchers are using experiments which ask participants to imagine how they would feel in other’s shoes in a real situation, instead of imagining a fictitious situation. It indicates a limitation in the current tests of vicarious cognitive dissonance, as nearly all studies are conducted with the need to imagine a situation and reflect on how one feels regarding this situation, instead of living it. Thus, the applicability or scaling potential of vicarious cognitive dissonance is unknown.

Our exploratory moderators’ results show the important variability in the study of cognitive dissonance. For example, we found at least 12 “topics” used as speech or writing materials. We also found that nearly half of the studies are on the induced-hypocrisy paradigm, and half on the induced-compliance paradigm. Most effects (*k* = 85) are studies in the laboratory, a few are studied online (*k* = 17), and none are studies in real settings. Finally, vicarious cognitive dissonance is a living field of research in the United States (*k* = 68) and France (*k* = 28), with minimal participants from other countries.

### Limitations

A first limitation lies in the number of meaningful moderators to improve the theory of vicarious cognitive dissonance. The lack of standardization leads to have too few studies on the same moderator. For example, there are 12 different topics, and we cannot test the effect of a particular topic because, for most of them, they are only used in one single study. Another example concerns the type of paradigm: only one study used the free choice paradigm. A higher level of standardization in design and the use of the same protocols would be beneficial to find the optimal conditions to detect an effect of vicarious cognitive dissonance.

A second limitation is the lack of diversity in the core aspects of the research thematic. Many experiments (19/24) were conducted with a student population. Although cognitive dissonance theory assumes that it is a general process experienced by the majority of the population, the lack of studies conducted on a general population could be a problem. The same can be said for the setting of the studies, as none of them were conducted in the real-world. Most studies were conducted in the laboratory and used the imagination of the participants.

### Implications

The low power in the studies included, in addition to the variability in the designs and procedures, lead to one core implication: researchers need to standardize their practice. They need to test rigorously the key aspects and necessary conditions of vicarious cognitive dissonance (i.e., group membership, perception how being free to choose) while keeping less important aspects constant, such as the topic for the speech or type of groups. They should do so using power analysis to determine an adequate number of participants and pre-registration to lower the risk of bias, especially in the analysis plan. We still note that the power we calculated might be lower than expected, as our publication bias estimators indicated that the effect size might be overestimated. For a more robust approach, researchers can use the results from our induced-compliance paradigm meta-analysis (*g* = 0.15) or the induced-hypocrisy paradigm (*g* = 0.27). To help researchers increase the size of the sample, the field can benefit from multiple laboratories’ projects, as demonstrated by Vaidis et al. (2023) who studied personal cognitive dissonance in more than 50 laboratories around the world.

From a theoretical perspective, the results of this meta-analysis allow us to better understand the process by providing an overview of a set of studies with different conceptions (Siddaway et al., 2019). It raised several fundamental questions on cognitive dissonance theory: what situations enable vicarious cognitive dissonance to emerge? What factors are necessary to elicit dissonance? What are its cognitive, emotional, and behavioral outcomes for the individual? Why are individuals motivated to reduce vicarious cognitive dissonance? Clarifying these questions proves essential to avoid certain theoretical errors (Bran & Vaidis, 2022; Vaidis & Bran, 2018). Our meta-analysis contributes to assessing current knowledge on these subjects and provides some guidance on how the field can increase the reliability of the findings.

## Conclusion

Results of our meta-analysis show a small effect size of vicarious cognitive dissonance (*g* = 0.41; *SE* = .07, *p* <.001, 95% CI [0.27, 0.54]) across various cognitive and affective measures. Our analyses suggest that individuals who observe another person perform a counter-attitudinal act experience dissonance vicariously, which motivates them to reduce the dissonance, in numerous ways. Individuals exposed to a vicarious cognitive dissonance situation tend to judge the observed person as more hypocritical, to express a stronger group similarity, and to change their attitudes regarding the topic. The results regarding the moderators were limited by the lack of diversity and standardization in research practice. Several concerns were raised regarding the operationalization of the dissonance situations and of the measurement of dissonance outcomes. Most studies had low statistical power, and publication bias estimators estimated a lower effect size than our meta-analysis. We provide guidance to increase reliability of the results, such as increasing the sample size and standardizing the operationalization of the dissonance elicitation and of the outcome measures.

## Notes

1. By “presence of vicarious cognitive dissonance,” we mean the situation in which participants observe the realization of an inconsistent act by another person.

2. By “vicarious induced-compliance,” we mean the situation in which participants observe the realization of an inconsistent act by another person through the induced-compliance paradigm.

3. By “vicarious induced-hypocrisy,” we mean the situation in which participants observe the realization of an inconsistent act by another person through the induced-hypocrisy paradigm.
